# Dual role of piperine: Preserving the degree of conversion of resin cement on bleached enamel and exhibiting antimicrobial activity

**DOI:** 10.4317/jced.63386

**Published:** 2025-11-30

**Authors:** Taira Endi de Flaviano Albuquerque, Lara Rabelo Aragão, Wildson Max Barbosa da Silva, Guida Hellen Mota do Nascimento, Solange de Oliveira Pinheiro, Juliana Paiva Marque Lima Rolim, Bruna Marjorie Dias Frota de Carvalho, Diana Araújo Cunha, Amanda Ávila Queiroz Pereira, Jiovanne Rabelo Neri

**Affiliations:** 1 Master in Dental Sciences, University Center Christus, Fortaleza, Ceará, Brazil; 2 Graduate School of Biomedicine, University Center Christus, Fortaleza, Ceará, Brazil; 3 Graduate School of Chemistry, State University of Ceará, Fortaleza, Ceará, Brazil

## Abstract

**Background:**

Piperine piperine extracted from Piper nigrun has a significant potential for use in dentistry and other areas of medical sciences. Promising results concerning this agent in in vitro tests can influence the development of new drugs, medical products, and clinical procedures. The present study aimed to extract, characterize, and evaluate the antimicrobial and antioxidant effect of piperine derived from Piper nigrun.

**Material and Methods:**

Extraction of piperine was carried out using a reflux system and confirmed by UV-Vis spectrophotometry and nuclear magnetic resonance spectroscopy. Piperine was diluted in distilled water at 0.001%, 0.002%, and 0.004%. Antioxidant activity of piperine was carried out by analyzing the degree of conversion (DC) in situ of self-adhesive resin cement applied to enamel fragments bleached with 35% hydrogen peroxide. Meanwhile, the antimicrobial activity was assessed using the broth microdilution technique. Statistical analysis of the DC was evaluated with the ANOVA and Student-Newman-Keuls test.

**Results:**

The minimum inhibitory concentration (MIC), minimum fungicidal concentration (MFC) and minimum bactericidal concentration (MBC) were performed with Kruskal-Wallis test. Level of significance adopted was p&lt;0.05. The yield of piperine extraction was 92.86%. No statistical difference between DC values of the piperine groups (0.001%, 0.002% and 0.004%), without whitening group and withening + cemented after 7 days (p&gt;0.05). The whitening + immediate cementation group showed the lowest DC when compared to the other groups (p&lt;0.05). The MBC evidenced antimicrobial activity (p&lt;0.05) at a concentration of 0.004% in comparison with the negative control, without treatment, and the positive control, using chlorhexidine 0.12%. The MFC revealed a reduction in colonies at a concentration of 0.004% when compared to the other groups and controls (p&lt;0.05).

**Conclusions:**

Piperine exhibited antifungal and antibacterial properties and avoided the reduction in the degree of conversion of a self-adhesive resin cement applied on bleached enamel.

## Introduction

There is a growing scientific interest in bioactive molecules with the capacity to neutralize free radicals, particularly those derived from natural sources or generated through semisynthetic modification processes (1) Natural products constitute abundant reservoirs of bioactive compounds characterized by extensive chemical diversity and broad-spectrum biological activities. Among these, secondary metabolites are especially noteworthy due to their significant therapeutic potential and biomedical applications (2). Research and the use of new drugs, dietary supplements, and plant-based sanitary products have increased considerably in recent years. Piperine, a nitrogenous alkaloid derived from piperidine, is the main active component present in black pepper (Piper nigrun) (3 - 5). Studies have shown that this compound presents several pharmacological effects and excellent therapeutic properties, including antimicrobial and antioxidant activity, rendering it a substance of considerable potential in medical science research (4 , 5 - 7). Piperine has presented remarkable antibacterial activity against Gram-positive bacteria (Bacillus subtilis, Enterococcus faecalis, Staphylococcus xylosus, Staphylococcus aureus, and Staphylococcus epidermidis) and Gram-negative bacteria (Escherichia coli, Klebsiella pneumoniae, and Salmonella enterica) (6). The alkaloid also exhibited antifungal activity against Cladosporium cladosporioides and Cladosporium sphaerospermum (8). Although piperine has been extensively studied, knowledge regarding its activity against Streptococcus mutans and Candida albicans remains scarce. In addition, piperine also presents antioxidant activity, which prevents the occurrence of oxidative cell damage by inactivating free radicals and reactive oxygen forms (5). The potential antioxidant effects of piperine remain unexplored within Dentistry (9). Dentistry faces challenges in performing esthetic restorations immediately after tooth whitening (9). Post-whitening, various treatments like replacing old restorations, dental enhancements, and applying veneers are frequently carried out due to color discrepancies (10). Hydrogen peroxide breakdown produces oxygen that interferes with resin material polymerization by generating superoxide radicals, impeding the extension of their polymeric chains (11). Studies indicate that a safe interval to perform adhesive procedures after bleaching would be between one to three weeks (12 , 13). To prevent treatment delays, employing antioxidant agents post-whitening has been proposed, enabling immediate continuation of the rehabilitation process (14). Thus, the present study aimed to extract, characterize, and evaluate the antimicrobial and antioxidant effect of piperine derived from Piper nigrun. The study's null hypotheses were: 1) No statistical variance between groups in terms of degree of conversion; 2) No statistical distinction among groups regarding minimum inhibitory and fungicidal concentrations; 3) No statistical variance among groups concerning minimum inhibitory and bactericidal concentrations.

## Material and Methods

- Piperine extraction Fifty grams of Piper nigrum powder underwent extraction with 150 mL of ethyl acetate at 70ºC for 5 hours, followed by solvent removal using a rotary evaporator. The resultant residue was treated with alcohol and aqueous KOH solution, filtered, washed, and dried. Confirmation of piperine isolation involved melting-point testing, UV-Vis spectrophotometry, and NMR spectroscopy. Yield assessment relied on comparing the extracted piperine mass to the expected content in black pepper. Alkaloid purity was determined using thin-layer chromatography (14). - Preparation of the aqueous piperine solutions Piperine was weighed and then diluted in distilled water (pH = 7.55) utilizing a vortex to achieve piperine solutions at concentrations of 0.001%, 0.002%, and 0.004% (w/v). All aqueous piperine solutions were colorless. - Degree of conversion (DC) in situ The study protocol received approval from the Human Research Ethics Committee (protocol #3.666.589). Thirty enamel A2 composite resin blocks of 3 mm height were created, photoactivated, then sandblasted, cleaned, and treated with Single Bond Universal adhesive (3M ESPE, St. Paul, MN, USA). Eight caries-free human third molars were prepared into enamel specimens. The blocks and specimens were divided into groups based on treatments and cementation time (Table 1).

Table 1

All except one group were bleached using 35% hydrogen peroxide thrice for a total of 45 minutes. Enamel surfaces underwent etching, followed by treatment with different solutions and drying. RelyX U200 (3M ESPE, St. Paul, MN, USA) self-adhesive resin cement was applied, and after photoactivation, slices were obtained for analysis. The degree of conversion was evaluated using a Raman microspectrometer under specific conditions. - Antimicrobial activity assays - Determination of the minimum inhibitory concentration (MIC) and minimum fungicidal concentration (MFC) The study assessed piperine's antifungal activity and minimum inhibitory concentrations (MIC) against Candida albicans(ATCC 10231) using the CLSI-recommended broth microdilution technique (document M27-A2). Yeasts from Sabouraud Agar were transferred to liquid culture medium to test susceptibility to piperine. Microplates with 96 wells received inoculum, culture medium, and various piperine dilutions (0.00025% to 0.004%). Nystatin served as a positive control. Multiple controls ensured method validity. Following incubation at 35ºC for 24 hours, the lowest inhibitory concentration was determined via visual analysis and ELISA absorbance. For Minimum Fungicidal Concentration (MFC) determination, MIC samples were plated on Sabouraud Dextrose agar and incubated at 35ºC for 24 hours in triplicate across three experiments. - Determination of the minimum inhibitory concentration (MIC) and minimum bactericidal concentration (MBC) The study tested piperine's impact on Streptococcus mutans using the CLSI-recommended broth microdilution method. Bacteria from BHI agar was transferred to liquid medium and exposed to different piperine concentrations in microplates alongside controls, including chlorhexidine. After incubation at 37ºC for 24 h the lowest inhibitory concentration was visually determined. MIC samples were then evaluated for Minimum Bactericidal Concentration by seeding them on BHI blood plates and incubating them in triplicate across three experiments for 24 hours. - Statistical Analysis The MFC and degree of conversion analysis utilized SigmaStat 3.5 software, incorporating Shapiro-Wilk and Brown-Forsythe tests for data distribution and varianceequality assessment. MIC, MFC and MBC data underwent Kruskal-Wallis testing, while degree of conversion data was assessed with Analysis of Variance. Post-hoc comparisons utilized the Student-Newman-Keuls test, with significance set at p&lt;0.05.

## Results

**Figure 1:**
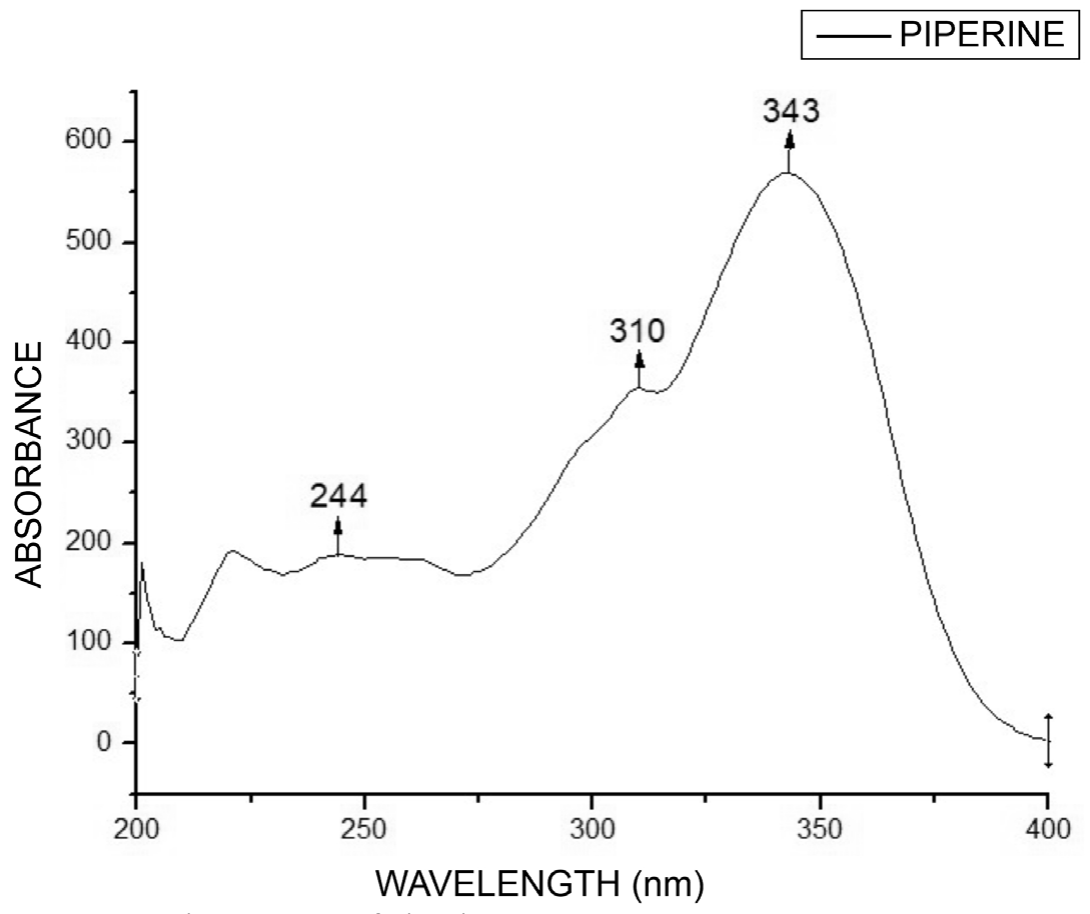
UV-Vis spectrum of piperine.

- Piperine extraction and characterization Using a reflux system, a crystalline yellow solid was extracted with a melting point of 125ºC. UV-vis spectroscopy in Figure 1 displayed a 343 nm wavelength, matching piperine.

1

After extraction, 78 mg of piperine was obtained, with a yield of 92.86% and purity of 99.9%. - Antioxidant activity test - Degree of Conversion in situ The data concerning the degree of conversion in situ are shown in Table 2.

Table 2

**Figure 2:**
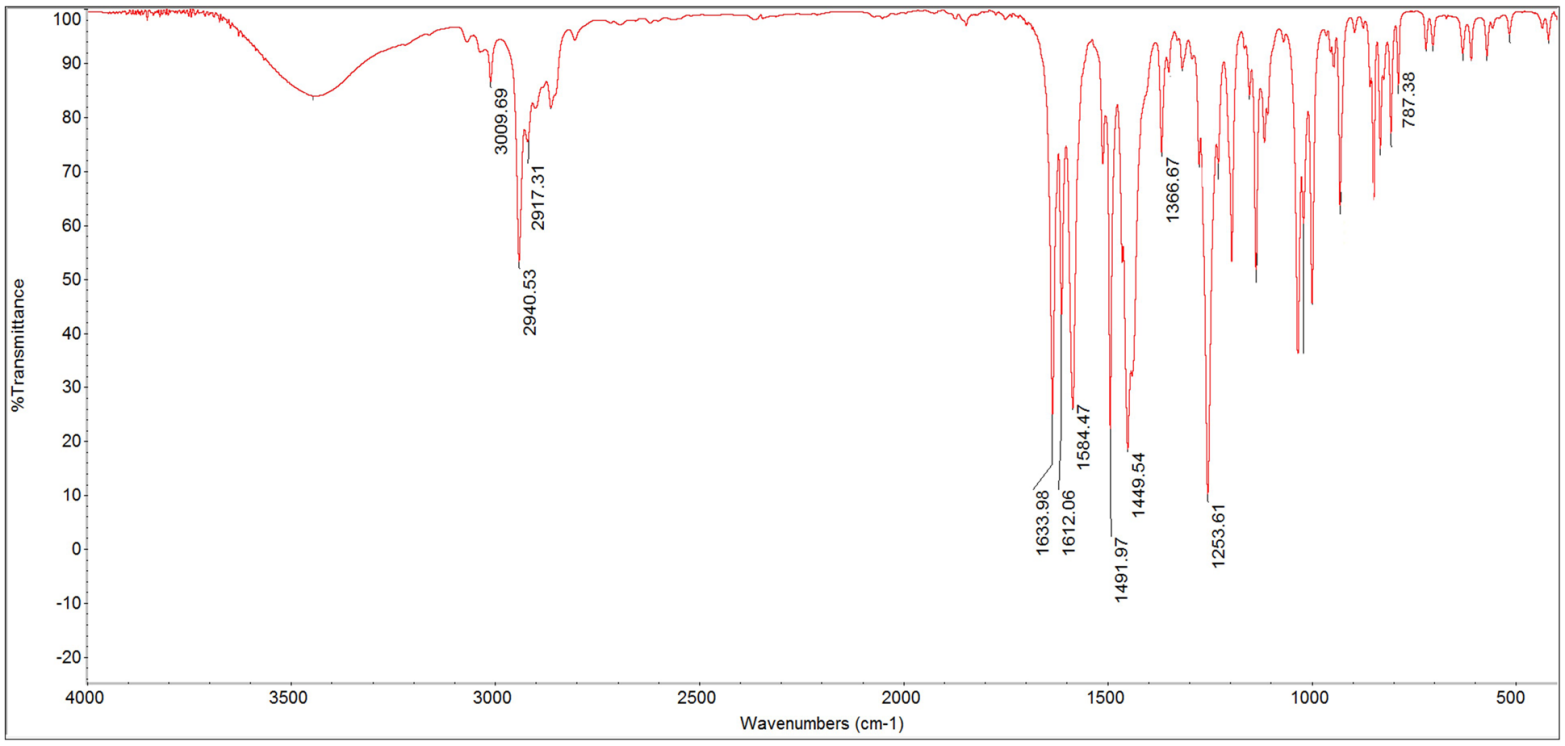
RMN spectrum of piperine.

There was no statistical difference between the groups without HP, HP + cementation after 7 days, HP + 0.001% piperine, HP + 0.002% piperine, and HP + 0.004% piperine (p&gt;0.05). - Determination of the minimum inhibitory concentration (MIC) and minimum fungicidal concentration (MFC) After performing the ELISA, a statistical difference was observed at concentrations 0.001%, 0.002%, and 0.004% in comparison with the negative and positive control (p&lt;0.05), as can be noted in Figure 2.

2

The MFC presented a reduction in colonies at the concentration 0.004% according to the CFU/mL count. - Determination of the minimum inhibitory concentration (MIC) and minimum bactericidal concentration (MBC) The MBC suggested antimicrobial activity (p&lt;0.05) at the concentration of 0.004% (8±0.2 CFU/mL) in relation to the negative control, the group without treatment (321 ± 2.5 UFC / mL), and the positive control containing 0.12% chlorhexidine (3.2±0.1 CFU/mL).

## Discussion

**Figure 3:**
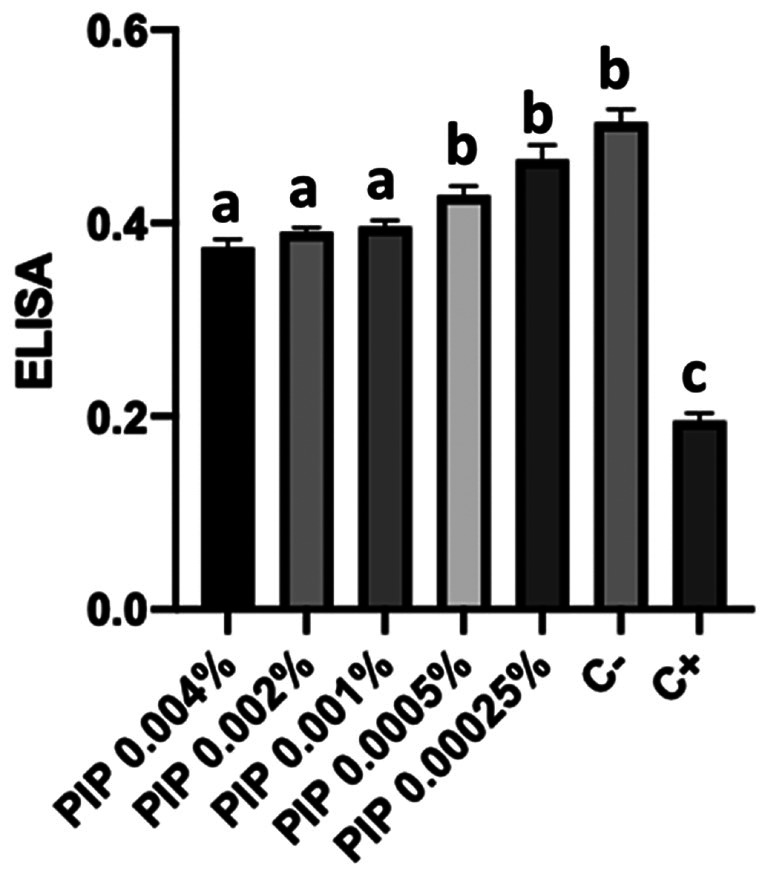
Therapeutic protocols performed in the MIC experiment and the numerical data obtained by the ELISA test. The height of the columns and the error bars indicate, respectively, the numerical data obtained by the ELISA test and the standard deviation. The groups identified using different letters denotes that there was a statistical difference between values (p<0.05).

The rejection of the first null hypothesis stems from a notable decrease in the degree of conversion seen in the self-adhesive resin cement applied immediately to whitened enamel in contrast to other groups, as indicated in Table 2. Research explores alternatives like enamel surface removal, alcohol pre-treatment, organic solvent-based adhesives, and antioxidants to enable immediate restorative procedures after whitening (15 , 16). Among the antioxidants tested in Dentistry, sodium ascorbate constitutes the most studied, and is considered by some authors as the best option to reestablish the decreased bond strength in bleached teeth (16 , 17). Nonetheless, the literature shows conflicting results hence the importance of seeking other options, among which is piperine (12 , 17). The study revealed that the degree of conversion of the self-adhesive resin cement applied to whitened enamel treated with various piperine concentrations mirrored unbleached specimens and those whitened and cemented after a week (Table 2), suggesting piperine potentially prevented reduction in conversion by inhibiting whitening-induced reactive oxygen species. Contrasting studies showed varied outcomes: glutathione and sodium ascorbate enhanced conversion, while alpha-tocopherol did not. Piperine's effectiveness was highlighted, even at lower concentrations and a short duration (60s), compared to antioxidants like sodium ascorbate, requiring a minimum 5-minute application. Moreover, piperine significantly reduced C. albicans colonies at 0.004% concentration (Fig. 3), rejecting the second null hypothesis and emphasizing the significance of antifungal agents in dentistry due to their role in common oral conditions like oral candidiasis and prosthetic stomatitis (18).

3

The research by Buitimea-Cantúa et al. (19) demonstrated significant antifungal activity of Piper nigrum and piperine against A. parasiticus, inhibiting radial growth notably at 200 mg/mL concentrations. MIC50 values for P. nigrum extract and piperine were 68 mg/mL and 67 mg/mL, respectively, with the extract exhibiting 78% bioactivity. Additionally, piperine showed moderate antifungal activity against A. flavus in Moon et al.'s study (20). Our study revealed complete inhibition of S. mutans growth at the highest piperine concentration (0.004%), rejecting the third hypothesis, consistent with findings from Deepak Dwivedi and Vinod Singh (21) on piperine's antibacterial and antibiofilm effects against S. mutans. D'Souza et al. (22) and Palaksha et al. (23) corroborated these results, indicating substantial antibacterial and antifungal activity of piperine against gram-positive bacteria like S. aureus and gram-negative bacteria. These findings underscore the potential of piperine in dentistry, despite its limited exploration in current literature, necessitating further investigation.

## Conclusions

Efficient extraction and characterization of piperine from Piper nigrum seeds showcased its promising antioxidant potential, preventing the reduction in self-adhesive resin cement's degree of conversion on hydrogen peroxide-bleached enamel. Furthermore, piperine demonstrated antifungal properties against C. albicans and bactericidal activity against S. mutans at a concentration of 0.004%.

## Figures and Tables

**Table 1:** Experimental design.

Groups	Description
Without HP (negative control)	Without whitening, without application of antioxidant agent (application of distilled water), and immediate cementation.
HP + immediate cementation	Whitening with hydrogen peroxide at 35%, without application of antioxidant agent (application of distilled water), and immediate cementation.
HP + cementation after 7 days (positive control)	Whitening with hydrogen peroxide at 35%, without application of antioxidant agent (application of distilled water) and cementation after 7 days.
HP + 0.001% piperine	Whitening with hydrogen peroxide at 35%, application of aqueous piperine solution at 0.001% w/v (during 60s), and immediate cementation.
HP + 0.002% piperine	Whitening with hydrogen peroxide at 35%, application of aqueous piperine solution at 0.002% w/v (during 60s), and immediate cementation.
HP + 0.004% piperine	Whitening with hydrogen peroxide at 35%, application of aqueous piperine solution at 0.004% w/v (during 60s), and immediate cementation.

1

**Table 2:** Degree of conversion values in situ (mean ± standard deviation), according to the applied treatment.

Groups (n=5)	Degree of conversion in situ
Without HP (control)	66.64±2.60A
HP + immediate cementation	61.01±1.98B
HP + cementation after 7 days	65.19±2.20A
HP + 0.001% piperine	64.80±1.36A
HP + 0.002% piperine	63.76±2.06A
HP + 0.004% piperine	64.67±1.76A

2

## Data Availability

The datasets used and/or analyzed during the current study are available from the corresponding author.
